# Time-Dependent Serum Brain-Derived Neurotrophic Factor Decline During Methamphetamine Withdrawal

**DOI:** 10.1097/MD.0000000000002604

**Published:** 2016-02-08

**Authors:** Wenwei Ren, Jingyan Tao, Youdan Wei, Hang Su, Jie Zhang, Ying Xie, Jun Guo, Xiangyang Zhang, Hailing Zhang, Jincai He

**Affiliations:** From the Department of Neurology, The First Affiliated Hospital of Wenzhou Medical University, Wenzhou (WR, JT, HS, JZ, YX, JH); Department of Psychiatry, Hangzhou Seventh People's Hospital, Hangzhou (YW); Sanyang Detoxification Institute, Wenzhou (JG); Beijing HuiLongGuan Hospital, Peking University, Beijing, China (XZ); Menninger Department of Psychiatry and Behavioral Sciences, Baylor College of Medicine, Houston, TX (XZ); and Department of Endocrinology, The First Affiliated Hospital of Wenzhou Medical University, Wenzhou, China (HZ).

## Abstract

Methamphetamine (METH) is a widely abused illegal psychostimulant, which is confirmed to be neurotoxic and of great damage to human. Studies on the role of brain-derived neurotrophic factor (BDNF) in human METH addicts are limited and inconsistent. The purposes of this study are to compare the serum BDNF levels between METH addicts and healthy controls during early withdrawal, and explore the changes of serum BDNF levels during the first month after METH withdrawal.

179 METH addicts and 90 age- and gender-matched healthy controls were recruited in this study. We measured serum BDNF levels at baseline (both METH addicts and healthy controls) and at 1 month after abstinence of METH (METH addicts only).

Serum BDNF levels of METH addicts at baseline were significantly higher than controls (1460.28 ± 490.69 vs 1241.27 ± 335.52 pg/mL; *F* = 14.51, *P* < 0.001). The serum BDNF levels of 40 METH addicts were re-examined after 1 month of METH abstinence, which were significantly lower than that at baseline (1363.70 ± 580.59 vs 1621.41 ± 591.07 pg/mL; *t* = 2.26, *P* = .03), but showed no differences to the controls (1363.70 ± 580.59 vs 1241.27 ± 335.52 pg/mL; *F* = 2.29, *P* = 0.13).

Our study demonstrated that serum BDNF levels were higher in METH addicts than controls during early withdrawal, and were time dependent decreased during the first month of abstinence. These findings may provide further evidence that increased serum BDNF levels may be associated with the pathophysiology of METH addiction and withdrawal and may be a protective response against the subsequent METH-induced neurotoxicity. Besides, these findings may also promote the development of medicine in the treatment of METH addiction and withdrawal.

## INTRODUCTION

The illegal psychostimulant, methamphetamine (METH), has been consumed by ∼25 million users worldwide.^1^ The wide availability, low cost, and long duration of psychoactive effects have made it a very desirable commodity among various populations. METH enables the abusers to experience a sense of alertness, increasing energy, and euphoria,^2^ but also leads to negative effects such as reductions in sustained attention, deficits in executive function, memory impairments, and neurochemical changes.^3,4^ The midbrain dopaminergic system was confirmed to play a critical role in the reward and addiction of METH.^5^ Besides, a large number of studies have conclusively demonstrated that dopamine (DA) is an important component of the mechanisms underlying METH neurotoxicity.^6–8^

Brain-derived neurotrophic factor (BDNF), a neurotrophin which is widely expressed in brain, plays an important role in maintaining the function and survival of DA neurons and protecting the DA neurons form toxic insults.^9,10^ Previous studies have demonstrated that BDNF was shown to be involved in the development of addiction.^11,12^ Meanwhile, several studies have also found that BDNF may interact with some of the effects induced by METH. For example, several studies in animals discovered that METH could increase striatal BDNF expression.^13,14^ Besides, studies by Dluzen DE et al found that alterations in striatal levels of BDNF reduced METH-induced neurotoxicity.^15,16^

Up to now, studies focusing on the relationship between METH abuse and BDNF in humans are limited and the results are inconsistent. The study by Chen PH et al found that serum BDNF levels declined during METH early withdrawal.^1^ Contrary to their results, Kim and his colleagues found that plasma BDNF levels in METH addicts remain elevated after 30 or more days of abstinence.^17^ Moreover, both these 2 studies were cross-sectional designed and the sample sizes of METH abusers were 59 and 50, respectively. Therefore, a follow-up study with a larger sample size is worthy of further doing to identify the relationships between METH abuse and BDNF levels. The purposes of this study are to identify the baseline differences in serum BDNF levels between METH addicts (179) and healthy controls (90), and the changes of serum BDNF levels in METH addicts (40) after 1 month of abstinence.

## METHODS

### Participants

A total of 179 METH addicts were recruited from Sanyang Detoxification Institute, located in Wenzhou, Zhejiang. Inpatients in this institute had no access to METH and were in rigid control of abstinence. All the patients were included in the study based on the following criteria: (1) age ≥ 18 years; (2) meet the Diagnostics and Statistical Manual of Mental Disorders, 4th edition (DSM-IV) criteria for current METH dependence;^18^ (3) positive urine test for amphetamine at admission; (4) METH abstinence for 1 to 7 days (period between last drug use and enrollment); (5) signed informed consent. Patients were excluded if they fulfill the criteria of DSM-IV for Axis I psychiatric disorder or substance dependence except METH or nicotine, were seropositive for HIV, or had serious medical illnesses that required pharmacological treatment. All the subjects did not take any medications during METH withdrawal.

We randomly selected 90 normal controls from the healthy subjects who went for physical examinations in the First Affiliated Hospital of Wenzhou Medical University. No self-reported family psychiatric history and no medication history were found in healthy controls. Clinical diagnosis was confirmed by board certified psychiatrists after interviews, and the study was approved by the Human Research and Ethics Committee of Wenzhou Medical University. All the subjects had written informed consent after a detailed description of this research.

### Measures

Demographic data was collected through the medical records and the self-reports. We also collected the information about the drug use, including the age at onset, average daily dose (in the past week), duration of drug use (the period between the first-ever METH use and most recent use), maximum daily dose, and route of drug administration. Withdrawal symptoms were assessed using amphetamine withdrawal questionnaire (AWQ).^19^ Craving was defined as a strong or intense desire^18^ and was assessed using a modified version of the visual analog scale (VAS), which is a reliable self-report instrument for assessing levels of craving at specific points in time, indicated on a scale from 0 (no craving) to 100 (most severe craving possible).^20^ The short form (13 items) of the Beck depression inventory (BDI)^21^ was used to measure self-reported depression symptoms. Each item is scored from 0 to 3 with a maximum combined score of 39. The Beck anxiety inventory (BAI)^22^ which consists of 21 items scored on a Likert-type scale from 1 to 4 was used to evaluate the severity of anxiety symptoms.

Serum BDNF levels were tested at baseline (abstinent for 1–7 days) in both METH addicts and healthy controls, and at 1-month endpoint in METH addicts. To reduce the possible rhythm variance bias, all the blood samples were collected between 8 and 10 AM. At baseline (with an average abstinent period of 3.60 ± 1.53 days), 179 METH addicts completed the measurement. Totally, 139 patients left the Sanyang Detoxification Institute for various reason such as discharge and referral before the 1-month endpoint (with an average abstinent period of 30.55 ± 2.20 days), and serum BDNF levels were reexamined in the remaining 40 METH addicts. Five milliliters of blood was collected and immediately centrifuged at 3500 rpm for 10 min. The serum obtained in this study was stored at −80°C before it could be thawed for assay.

Serum BDNF levels were tested by trained operators using the DuoSet ELISA Development System (Catalog number DY248, R&D Systems, USA) according to the manufacturer's instruction. The operators were blind to the study design. All the measurements were carried out in duplicate and expressed as pg/mL. The detection range of the assay was 20 to 4000 pg/mL. The intra-assay coefficients were <5%, whereas the inter-assay were <10%.

### Statistical Analysis

Demographic characteristics of the METH addicts and healthy controls were compared using Student's *t* test for continuous variables and chi-squared for categorical variables. The differences of serum BDNF levels between METH addicts and the healthy controls at baseline or 1-month endpoint were compared with 1-way analysis of variance (ANOVA). In addition, when there was a significant ANOVA, analysis of covariance was also adopted, with the age, gender, education, and body mass index (BMI) being included as the covariates. With paired *t* test, we analyzed the differences of serum BDNF levels between baseline and 1 month after METH withdrawal. Pearson correlation analysis or Spearman correlation analysis was used according to the data distribution to examine the relationships between serum BDNF levels and other variables.

Statistical analyses were performed in IBM SPSS software (ver. 16.0; SPSS Inc., Chicago, IL). All *P* values were 2 tailed with the significance level set at 0.05.

## RESULTS

### Characteristics of METH Addicts and Healthy Controls

One hundred and seventy-nine METH addicts and 90 healthy controls were recruited. The demographic and clinical data of METH addicts were summarized in Table 1. Education years in METH addicts were significantly lower than that of healthy controls (*P* < 0.001). No differences were found in any other variables between METH addicts and healthy controls. In addition, compared to the 139 patients leaving the Sanyang Detoxification Institute before 1-month endpoint, the remaining 40 patients had an older mean age (34.18 ± 7.67 vs 30.17 ± 7.64 years; *F* = 8.50, *P* = 0.004). No differences were found in any other factors.

**TABLE 1:**
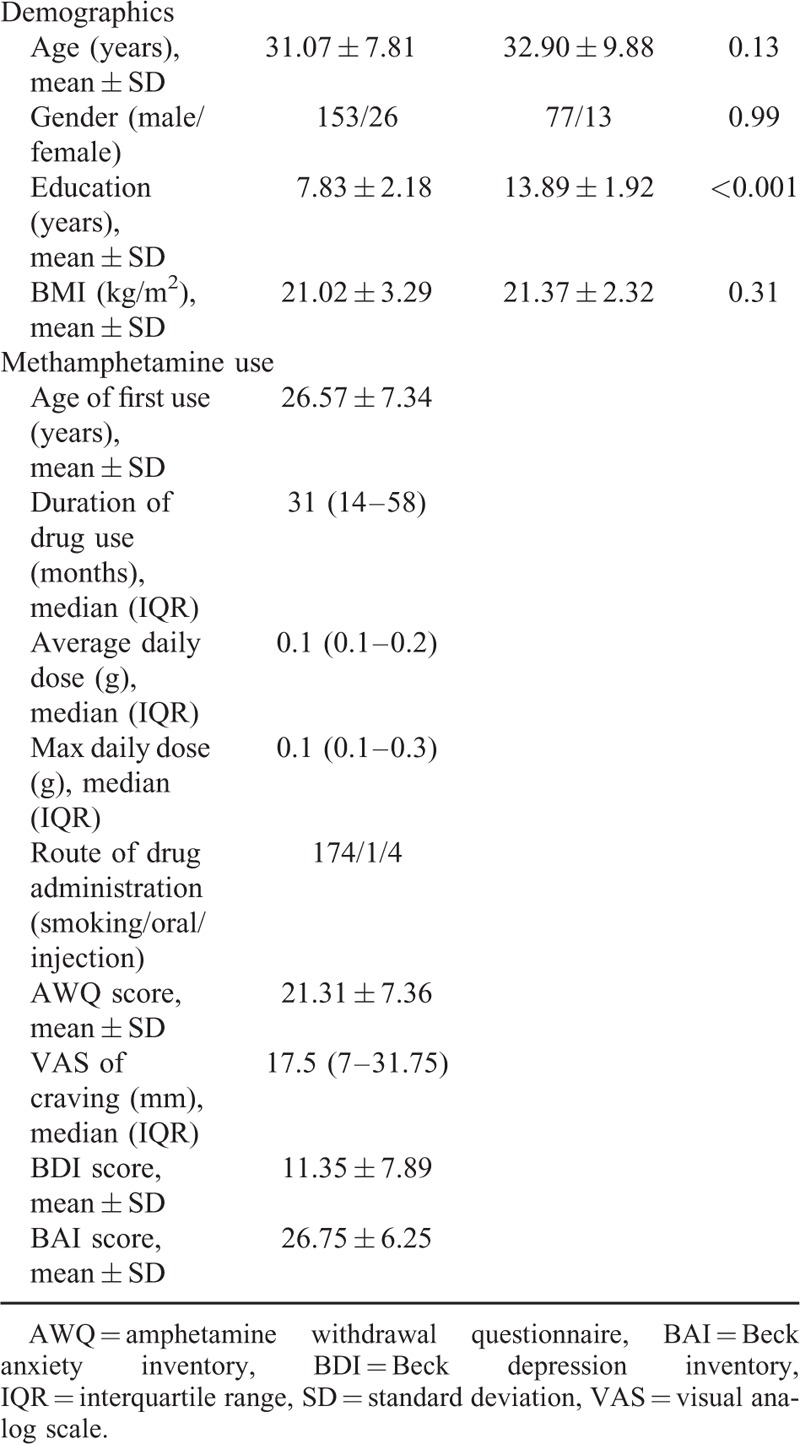
Characteristics of Methamphetamine Addicts and Healthy Controls

### Serum BDNF Levels of METH Addicts and Healthy Controls

**FIGURE 1:**
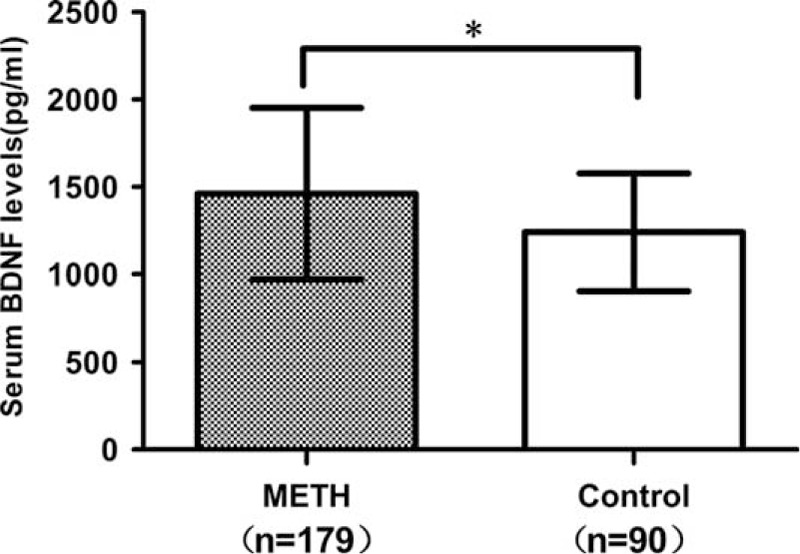
Comparison of the serum BDNF levels between METH addicts and healthy controls at baseline. An asterisk (^∗^) indicates significant difference between these 2 groups (*P* < 0.001). BDNF = brain-derived neurotrophic factor, METH = methamphetamine.

As was shown in Figure 1, the serum BDNF levels of METH addicts were significantly higher than that of healthy controls at baseline (1460.28 ± 490.69 vs 1241.27 ± 335.52 pg/mL; *F* = 14.51, *P* < 0.001). The results remain unchanged even after controlling for the age, gender, education, and BMI (*F* = 5.33, *P* = 0.02).

**FIGURE 2:**
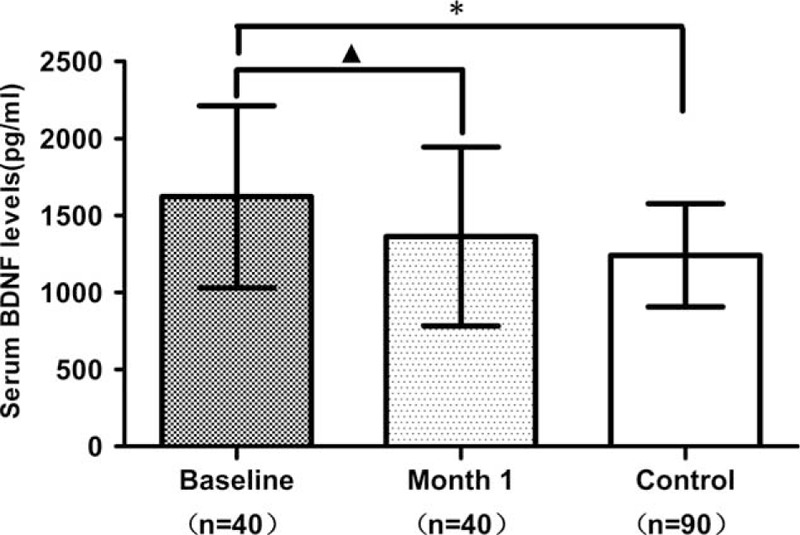
Comparison of the BDNF serum levels between METH addicts and healthy controls at baseline and 1-month endpoint. Values are expressed in pg/mL. A triangle (Δ) indicates significant difference between the groups (*P* < 0.05). An asterisk (^∗^) indicates significant difference between the groups (*P* < 0.001). BDNF = brain-derived neurotrophic factor, METH = methamphetamine.

As was shown in Figure 2, the baseline serum BDNF levels of the 40 METH addicts were significantly higher than that at 1-month endpoint (1621.41 ± 591.07 vs 1363.70 ± 580.59 pg/mL; *t* = 2.26, *P* = 0.03), as well as the healthy controls (1621.41 ± 591.07 vs 1241.27 ± 335.52 pg/mL; *F* = 21.66, *P* < 0.001). However, the serum BDNF levels at 1-month endpoint showed no differences to the healthy controls (1363.70 ± 580.59 vs 1241.27 ± 335.52 pg/mL; *F* = 2.29, *P* = 0.13).

### Serum BDNF Levels and Clinical Characteristics

In healthy controls, no correlations were found between the serum BDNF levels and any other variables, including the age (*r* = −0.141, *P* = 0.19), BMI (*r* = −0.065, *P* = 0.54), and education (*r* = 0.038, *P* = 0.72). Besides, the men showed no difference to the women in serum BDNF levels (1226.57 ± 338.14 vs 1328.34 ± 318.08 pg/mL; *F* = 1.023, *P* = 0.31).

In METH addicts, higher serum BDNF levels were correlated with older age (*r* = 0.157, *P* = 0.04). However, we did not find any correlations between serum BDNF levels and education (*r* = −0.027, *P* = 0.72), BMI (*r* = −0.100, *P* = 0.19), average daily dose (*r* = −0.059, *P* = 0.52), max daily dose (*r* = −0.019, *P* = 0.83), age of first use (*r* = −0.075, *P* = 0.32), duration of METH use (*r* = −0.118, *P* = 0.12), withdrawal symptoms (*r* = −0.020, *P* = 0.80), craving (*r* = 0.040, *P* = 0.75), BDI (*r* = 0.50, *P* = 0.53), and BAI (*r* = 0.23, *P* = 0.77). Besides, the men showed no difference to the women in serum BDNF levels (1445.32 ± 493.35 vs 1548.32 ± 474.42 pg/mL, *F* = 0.979, *P* = 0.32).

## DISCUSSION

To the best of our knowledge, this is by far the first follow-up study to demonstrate a time-dependent decrease of serum BDNF levels in METH addicts during the first month of withdrawal. Specifically, there are 2 major findings in present study. First, we found that METH addicts had significantly higher serum BDNF levels than healthy controls at baseline. Second, the baseline serum BDNF levels declined significantly after 1 month of METH abstinence.

Previous studies have discovered that METH could increase BDNF expression in various brain areas.^14,23^ Besides, in METH addicts, plasma BDNF levels remain elevated after 30 or more days of METH abstinence.^17^ Moreover, the present study found that METH addicts had significantly higher serum BDNF levels than healthy controls during early withdrawal. These findings suggested that BDNF may be involved in the process of METH addiction and withdrawal. However, the mechanisms underlying this association between increased BDNF levels and METH use were still not very clear. It was hypothesized that the increased BDNF levels may offer neuroprotective potential against METH-induced neurotoxicity. It has been well established that the rewarding effects of METH are mainly mediated through its effects on the mesolimbic dopaminergic system.^24^ The abuse of METH can promote the release of dopamine and is toxic to the dopamine neurons, which eventually decreases the synaptic dopamine levels.^25,26^ Neuroimaging studies in abstinent human METH addicts had observed that METH could lead to persistent decreases in the levels of brain DA, and dopamine transporters density (DAT) in various brain areas.^27^ Besides, repeated amphetamine administration could produce nigrostriatal dopaminergic neurotoxicity,^28^ and METH could reduce the dopaminergic cells in the substantia nigra.^29^

BDNF can modulate DA levels, contribute to the survival and maintenance of DA neurons, and alter neurotransmitter synthesis, metabolism, and release.^30^ A study by Narita M et al found that intranucleus accumbens infusion of BDNF could reduce dopamine release and dopamine-related behaviors induced by methamphetamine.^31^ In addition, several studies in animals showed that alterations in striatal levels of brain-derived neurotropic factor (BDNF) reduced METH-induced neurotoxicity.^15,16^ Moreover, BDNF could dose-dependently block the METH-induced neuronal cell death in vitro and altered BDNF expression could reduce METH induced striatal dopamine depletion in mice.^32^ Taken together, the increased BDNF levels following METH may be a protective response against the subsequent METH-induced neurotoxicity, and DA may be an important mediating factor that involved in the regulation of BDNF expression following exposure to METH.

To date, studies investigating the serum BDNF levels among METH addicts were limited. A study by Kim et al found that chronic METH addicts who had been abstinent for at least 30 or more days had significantly higher plasma BDNF levels than healthy controls,^17^ which was consistent with our results at baseline. Meanwhile, another study in human by Chen et al found that serum BDNF levels were significantly lower in the 59 cases of METH addicts during early withdrawal (abstinent for ≤ 21days) compared to healthy controls.^1^ In the present study, the serum BDNF levels were significantly higher than healthy controls at baseline (abstinent for 1–7days). These differences may be due to various factors, such as days of abstinence and sample size. In addition, the present study also found that the serum BDNF levels declined significantly during the first month of METH abstinence, and showed no difference to the controls at 1-month endpoint, which was somewhat different from the study by Kim et al. Several factors may account for this difference. For example, in the present study, the serum BDNF levels of the METH addicts at 1 month were compared to the baseline levels of controls, which may affect the results to some extent. In addition, only male patients were included in the study by Kim et al. The time-dependent decrease of serum BDNF levels should also be noted. As the studies by Kim et al and Chen et al only test the BDNF levels once during early withdrawal, we were unable to see whether the time-dependent decrease of BDNF also existed in their studies. Of note, several recent studies in animal and human had drawn similar results to the present study. A study in rats by McFadden LM et al found that at 2 and 24 h after cessation of METH self-administration, BDNF protein levels were significantly increased by 89% and 63%, respectively, compared to yoked saline controls. However, after 1 month of abstinence, BDNF protein levels were significantly decreased. They also found that BDNF mRNA expression was increased 2 h after cessation of METH self-administration but returned to control level at 24 h and 1 month.^33^ Furthermore, a study by Hilburn et al found that serum BDNF levels were negatively correlated with the number of abstinent days in human psychostimulant users.^34^ Given the above, it can be concluded that BDNF may play a role in METH withdrawal and may serve as a candidate biomarker for METH withdrawal.

In present study, we also observed that older patients showed higher serum BDNF levels among METH addicts. Several recent studies had also reported similar results. For instance, both Bus B and Elfving B had in their studies found an age-dependent increase in serum BDNF levels for women, but not men.^35,36^ However, most of the patients were men in the present study. Therefore, further research with equal proportion of men and women would be necessary to clarify the effects of age and gender on the serum BDNF levels.

There are several limitations in the present study. First, the BDNF levels in our study were measured in serum but not brain, which may impact the results to some extent. However, previous studies demonstrated that peripheral blood levels of BDNF were related to central BDNF expression.^37,38^ Besides, there was evidence that BDNF could pass through the blood-brain barrier via a high-capacity, saturable transport system.^39^ Therefore, the serum BDNF levels could, at least partly, reflect the brain BDNF levels. Future studies focusing more attention on the brain BDNF levels would be of high value. Second, owing to the frequent discharge and usual referral, only 40 patients finished the measurement of BDNF at 1 month. A higher follow-up rate will be necessary in the future studies to further elucidate the association between serum BDNF levels and the length of abstinence. Third, the serum BDNF levels of the healthy controls at 1 month were not examined, which may affect the outcomes to some extent. Fourth, the 40 patients finishing the 1 month follow-up were older, which may bring some biases to this study. Despite of these limitations, some strengths of this research cannot be ignored. For example, the initial large sample size (179) of our study makes the results more convincing. Besides, the follow-up design of this research is of more value in elucidating the relationship between the BDNF levels and abstinent days in METH addicts.

In summary, our study demonstrated that serum BDNF levels were increased in METH addicts during early withdrawal and were time-dependent decrease during the first month of abstinence. These findings may provide further evidence that increased serum BDNF levels may be associated with the pathophysiology of METH addiction and withdrawal and may be a protective response against the subsequent METH-induced neurotoxicity. Besides, these findings may also promote the development of medicine in the treatment of METH addiction and withdrawal.
